# Applying Acoustic Emission Technique for Detecting Various Damages Occurred in PCL Nanomodified Composite Laminates

**DOI:** 10.3390/polym13213680

**Published:** 2021-10-26

**Authors:** Ali Gholizadeh, Hasan Mansouri, Ali Nikbakht, Hamed Saghafi, Mohamad Fotouhi

**Affiliations:** 1 Department of Mechanical Engineering, Amirkabir University of Technology, Tehran 1591634311, Iran; aligholizade@aut.ac.ir; 2 Aerospace Engineering Department, K. N. Toosi University of Technology, Tehran 1591634311, Iran; h.mansoori@email.kntu.ac.ir; 3 New Technologies Research Center (NTRC), Amirkabir University of Technology, Tehran 1591634311, Iran; saghafi@tafreshu.ac.ir; 4 Department of Mechanical Engineering, Tafresh University, Tafresh 7961139518, Iran; 5 School of Engineering, University of Glasgow, Glasgow G12 8QQ, UK

**Keywords:** composite laminates, PCL nanofibers, fracture test, acoustic emission technique

## Abstract

Interleaving composite laminates by nanofibers is a well-known method of increasing interlaminar fracture toughness. Among many possibilities, polycaprolactone (PCL) nanofibers is one of the best choices for toughening composite laminates. The influence of PCL on delamination mode of failure is considered before. However, the effect of PCL on other damage modes, such as fiber breakage and matrix cracking, is yet to be studied. In this study, the acoustic emission (AE) technique is applied to determine the effect of toughening composite laminates by PCL nanofibers on matrix cracking, fiber/matrix debonding, and fiber breakage failure mechanisms. For this purpose, mode I and mode II fracture tests are conducted on modified and non-modified glass/epoxy laminates. Three different methods, i.e., peak frequency, wavelet transform, and sentry function, are utilized for analyzing the recorded AE data from mode I test. The results show that applying PCL nanofibers not only increases the mode I critical strain energy release rate by about 38%, but also decreases different failure mechanisms by between 75 and 94%.

## 1. Introduction

Nowadays, laminated composite parts are extensively used in engineering structures since they have desirable mechanical properties such as high specific stiffness and strength. On the other hand, various damage modes occur in these materials, e.g., fiber/matrix debonding, matrix cracking, fiber breakage, and delamination can restrict their applications [1,2].

Among these failure modes, removing or at least decreasing the delamination problem attracts the attention of more researchers. One of the effective ways to reduce or delay the delamination is to apply nanofiber mats between the composite layers [3,4]. The considerations prove that the nanofibers can increase the interfacial fracture toughness, strength, and resistance to delamination under static, fatigue, and impact loadings. This fact is due to the unique properties of these nanofiber mats, such as high surface-to-volume contact ratio, flexibility, and suitable mechanical properties [5]. 

The first research on the effect of applying nanofibers between composite layers was carried out by Dizenis and Ranker [6]. They showed that the use of nanofibers between layers of composite parts increases interlayer toughness, strength, and delamination resistance without significantly reducing the sheet properties of the layers and increasing structural weight. 

Electrospinning is a well-known method of producing nanofiber mats which are interleaved between composite layers for increasing interlayer toughness, strength, and delamination resistance. Saghafi et al. have used electrospinning to produce interlaminar 66 nylon, polycopperlatone (PCL), and a combination of these two nanofiber mats. They have investigated the energy reduction rate of mode I and mode II in their study [7]. Zhang et al. [8] have conducted a research to obtain suitable parameters for electrospinning nanofibers of PCL, polyvinylidene fluoride (PVDF), and polyacrylonitrile (PAN). They have used mechanical tests such as mode I test to obtain mechanical properties and to prove the efficiency of applying nanofibers in increasing the interfacial fracture toughness of mode I.

Although delamination is a common failure mechanism in composite structures, other damage mechanisms such as matrix cracking, fiber/matrix debonding, and fiber breakage can similarly significantly affect the functionality aspects of the structure. Interleaving nanofiber mats between the layers of a composite structures not only decreases the delamination (and thus increases interlayer toughness), but also decreases other failure mechanisms. Thus, it can be beneficial to investigate the effect of nanofibers on failure mechanisms other than delamination. 

Acoustic emission (AE) is a non-destructive technique that can be used to investigate the failure mechanisms in laminated composite structures [9]. AE signals are high frequency sound waves which are produced as a result of strain energy release due to internal events such as damaged mechanisms during the deformation of a material or a structure [10]. The recorded AE signals may be analyzed to investigate and to determine different failure mechanisms [11]. Most of AE, such as peak frequency, counts, energy, and amplitude, are related to user-defined parameters such as hit definition time, peak definition time, and threshold level, which, by using AE waveform-based analyzing methods such as wavelet transform and Shannon’s entropy, can be reduced to the effect of user-defined parameters [12]. Saeedifar et al. [11] have utilized the AE method to investigate the behavior of delamination propagation and to evaluate the critical value of the strain energy in mode I, mode II, and mixed mode tests. In order to identify interlaminar fracture toughness, they have used ASTM standard methods, FEM analysis, the AE method, and sentry function. They showed that the G_C_ values obtained by the sentry function method and FEM analysis are in a close agreement with the results of nonlinear methods, which are recommended in the ASTM standards. Gholizadeh et al. [3] utilized AE method for considering the effect of Nylon 66 on different damage modes under mode I and mode II loadings. For analyzing AE signals, they have used a peak frequency-based method and wavelet transform. AE analysis showed the significant reduction of different damage modes in the nanomodified laminates.

As mentioned, determining the effect of nanofibers on different failure mechanisms is an essential step in designing composite materials. Thus, in this study, PCL elecrospun nanofiber mats were interleaved between glass/epoxy composite layers, and the effect of nanofibers on matrix cracking, fiber/matrix debonding, and fiber breakage failure mechanisms was investigated by means of AE. In order to achieve this, mode I and mode II fracture tests were carried out and AE signals were recorded during mode I tests. Three methods, including the conventional method, Wavelet Packet Transform (WPT), and sentry function, were used for analyzing AE signals and determining the effect of interleaving nanofibers on different damage mechanics.

## 2. Materials and Methods

### 2.1. Materials

In order to prepare the test specimens, unidirectional prepreg glass/epoxy composite layers (Metal T.I.G. Company, Bologna, Italy) with a glass fiber density of 1200 g/m^2^ were used. The weight percentage of epoxy resin of these prepreg layers was equal to 32%, while the approximate thickness and the density of each layer were 0.865 mm and 1765 gr/m^2^, respectively.

As mentioned, PCL nanofiber mats (University of Bologna, Bologna, Italy) are considered in this study. In order to produce the nanofiber mat, laboratory PCL polymer was used where a combination of formic and acetic acids was used as the solvent.

### 2.2. Producing of Nanofiber Mats

**Figure 1:**
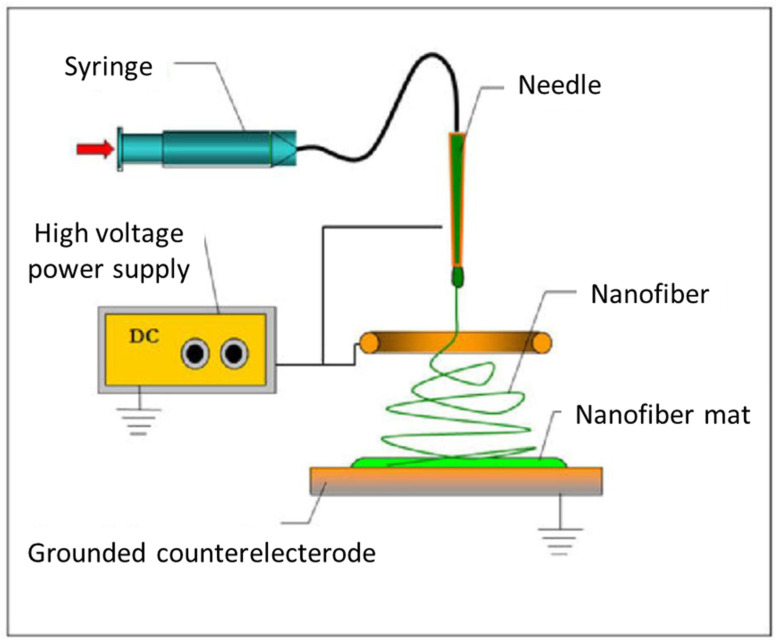
Schematic diagram of electrospinning process.

Electrospinning is an advanced method that is widely utilized in producing nanofiber mats. In this method, a high voltage power supply was used to generate an electric field between the flow of a polymeric solution (which contains a dissolved or melted polymer) from a micro-diameter nozzle and a conductive collector. This field was created by connecting the polymeric solution to one of the electrodes of a high voltage power supply, while the other electrode is connected to the conductive collector. The polymeric solution was pumped into a syringe which needle acts as the nozzle. As the voltage increases, the electric field, or electrostatic repulsion, overcomes the surface tension of the polymer, and the charged jet exits from the syringe tip. At this threshold voltage, the hemispherical surface of the solution (in the capillary tube) needle was stretched to form a structure called the Taylor cone [13]. A schematic diagram of electrospinning process is shown in Figure 1. In the electrospinning process, the diameter and the distribution of nanofibers are controlled by the voltage and the injection rate of the solution. The result of such a process is a mat which consists of the nanofibers of the dissolved polymer.

In this study, formic and acetic acids were used as the solvent of the PCL polymer. The weight percentages of the formic and acetic acids in the solvent were 60 and 40 percent, respectively. The solvent was then used to produce the polymeric solution by adding 15 weight percentage of PCL [14]. In order to completely and uniformly dissolve the polymer particles, the solution was stirred by a magnetic stirrer for 12 h.

**Figure 2:**
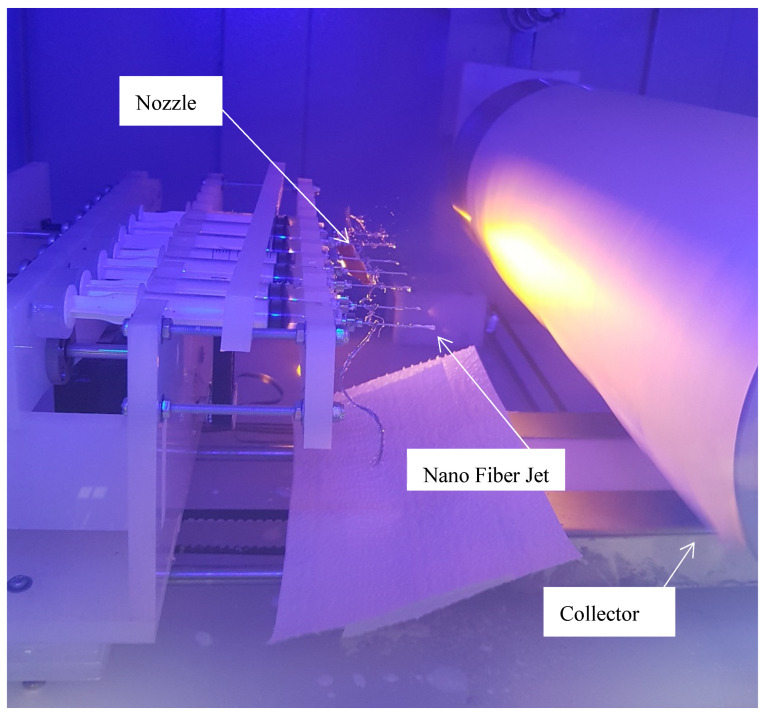
Nanofiber electrospinning process.

**Figure 3:**
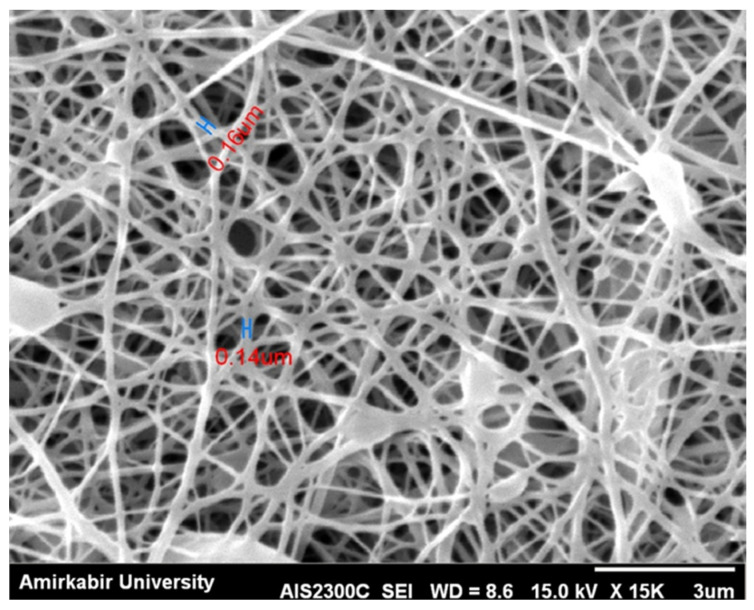
Produced nanofibers surface taken by SEM process.

In order to produce the nanofiber mat, the rotational speed of the collector and the injection rate at each nozzle were set to 100 rpm and to 0.9 mL/h, respectively [15]. Moreover, the applied voltage between the nozzle and the collector was considered to be 17 kV, and the distance between the nozzle tip and the collector surface was set to 10 cm. Accordingly, a jet of nanofibers is formed at the tip of the nozzle, and then the nanofibers are thrown to the collector surface. The electrospinning process is demonstrated in Figure 2. After 8 h of injection, a nanofiber mat with a mean thickness of 70 microns was obtained. The mean diameter of the nanofibers of this mat was 150 nanometers. Figure 3 shows an SEM (JEOL (Europe) BV, Nieuw-Vennep, The Netherlands) image of the nanofiber surface.

### 2.3. Production of Test Specimens

Modes I and II test specimens are made by ASTM D5528 [16] and ASTM D7905 [17] standards requirements, respectively. All specimens consisted of four prepreg glass/epoxy layers. The dimensions of the specimens were 170 × 3.5 × 20 mm^3^, and nanofiber mats were placed between the interface of the layers in the direction of the length of the specimen. To create an initial crack, a layer of fireproof fabric with a length of 60 mm was placed at the edge of the specimen, between the second and third layers (which is the mid interlayer). In order to investigate the effect of PCL nanofibers in some of both the mode I and mode II test specimens, a layer of PCL nanofiber was placed along the initial crack; thus, the nanofiber mats were only applied to the mid-interlayer of the specimen. Hereafter, these specimens are called nanomodified, while the specimens without nanofibers are referred to as reference samples. 

### 2.4. Test Procedure

Modes I and II tests are conducted by means of an HIWA universal test machine (HIWA, Tehran, Iran). In order to record the applied load during the mode I and mode II tests, a tensile Bongshin S-shaped load cell of 500 kg capacity was used. 

Mode I tests were performed based on the recommendations of ASTM D5528 [16] standard, where the speed of the applied load is set to be equal to 3 mm/min. In addition, two hinges were glued on both sides of each test sample at a distance of 30 mm from the crack tip in order to apply load to the samples. In order to increase the strength of adhesive connection, the surface of hinges and composite samples were roughed with a low mesh size grind. According to ASTM standard, there are three methods for identifying the load and displacement that caused delamination onset. Visual inspection was applied in this study. The critical strain energy release rate is calculated by Equation (1):

(1) $$G_{IC}=\frac{3P\delta }{2B(a+\Delta )}$$

where *P* is the applied load, *δ* is the load point displacement, *B* is the specimen’s width, *a* is the pre-crack length, and ∆ is the crack growth [16].

**Figure 4:**
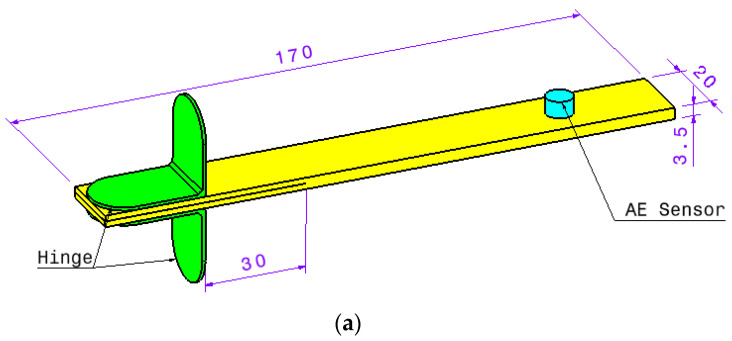

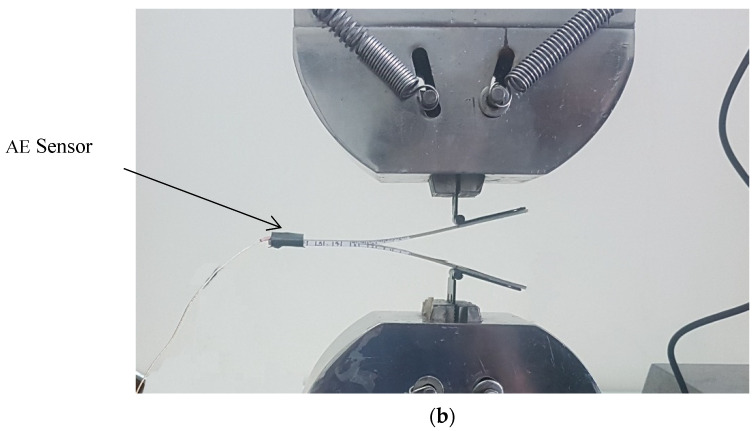
(**a**) Mode I test dimension (**b**) test setup.

During mode I tests, a wideband AE sensor with a resonance frequency of 513.25 KHz and optimum operating range between 100 and 750 KHz was placed at a distance of 80 mm from the initial crack tip to record the AE signals that generated during mode I tests. In addition, for enhancing the energy of recorded AE signals, we used a 2/4/6-AST preamplifier with a gain selector of 40 dB. The sampling rate of the AE data was considered to be 2 MHz, and the threshold of the recording AE signals was set equal to 38 dB. The dimension of these samples and mode I test setup are shown in Figure 4.

**Figure 5:**
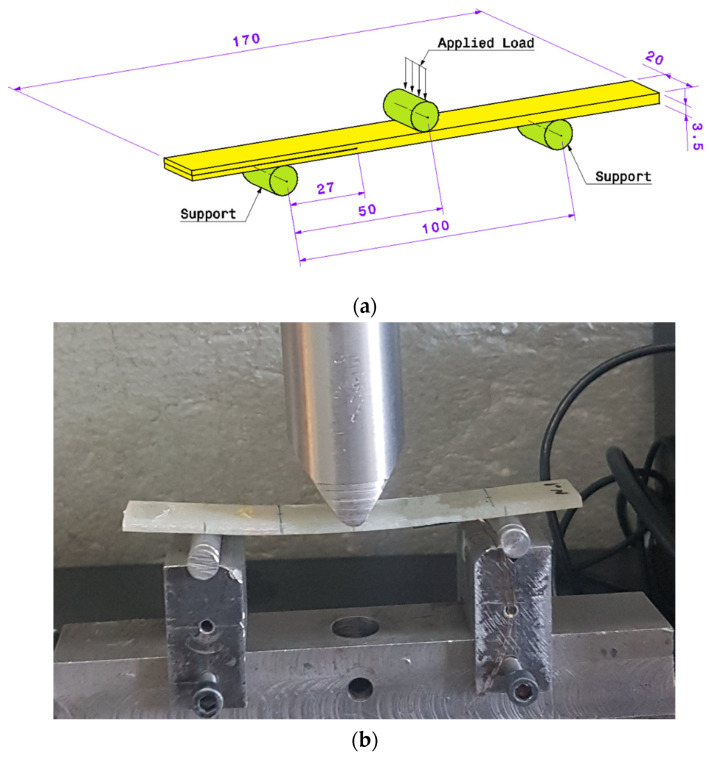
(**a**) Mode II tests dimension (**b**) test setup.

Mode II tests are also performed according to ASTM D7905 [17] standard. Figure 5a schematically shows the dimensions of the of the test setup. As can be seen in the figure, the distance between the two supports is considered to be equal to 10 cm. The force is exerted to the specimen exactly at the middle of the distance between the two supports (5 cm from each support). The distances of the initial crack tip from each of the supports are 27 mm and 73 mm, and the applied load speed is set equal to 1 mm/min. The mode II test setup is shown in Figure 5b.

### 2.5. Acoustic Emission Analysis

**Figure 6:**
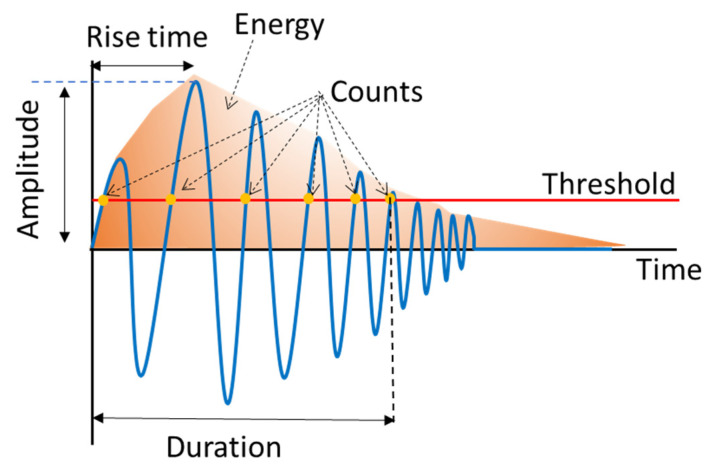
AE signals parameters.

**Figure 7:**
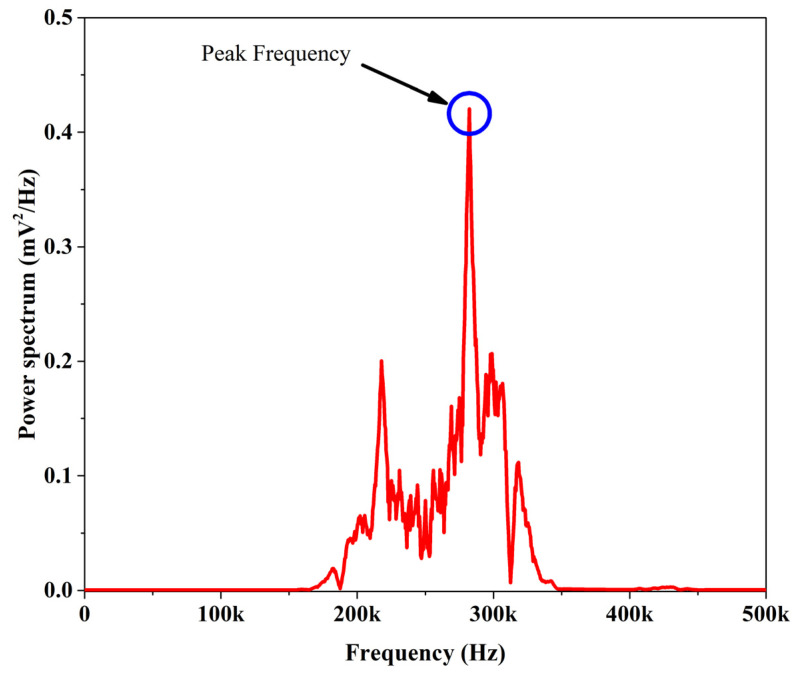
The frequency spectrum of an AE signal.

In this study, AE analysis was utilized with the aim of investigating the effect of including nanofibers on the failure mechanisms that occurs during mode I tests. Three methods, including conventional method, Wavelet Packet Transform (WPT), and sentry function, were used for analyzing AE signals. Each of recorded AE signals was considered to be caused by only one damage mechanism in the conventional method, while, in WPT method, each AE signal was assumed to be caused by a combination of damage mechanisms. In the conventional method, the parameters of an AE signal, such as amplitude, count, energy, rise time, duration, and peak frequency, were used; these parameters are shown in Figure 6. The peak frequency is one of these parameters that was used in this method, intensively. In order to calculate the peak frequency, two steps should be considered and could not be achieved from the waveform directly. In the first step, different ranges of the recorded AE signal frequencies are determined by means of Fast Fourier Transform (FFT). The frequency spectrum of an AE signal that was calculated with the FFT method is shown in Figure 7. The second step involves considering the frequency accompanied with the maximum energy as the peak frequency of the signal (Figure 7). According to the results obtained by Mohammadi et al. [18], different ranges of the peak frequencies are related to different failure mechanisms, as shown below:

Peak frequencies between 62.5 and 250 kHz are related to matrix cracking;

Peak frequencies between 250 and 375 kHz shows fiber/matrix debonding;

Peak frequencies between 375 and 500 kHz refers to fiber breakage.

**Figure 8:**
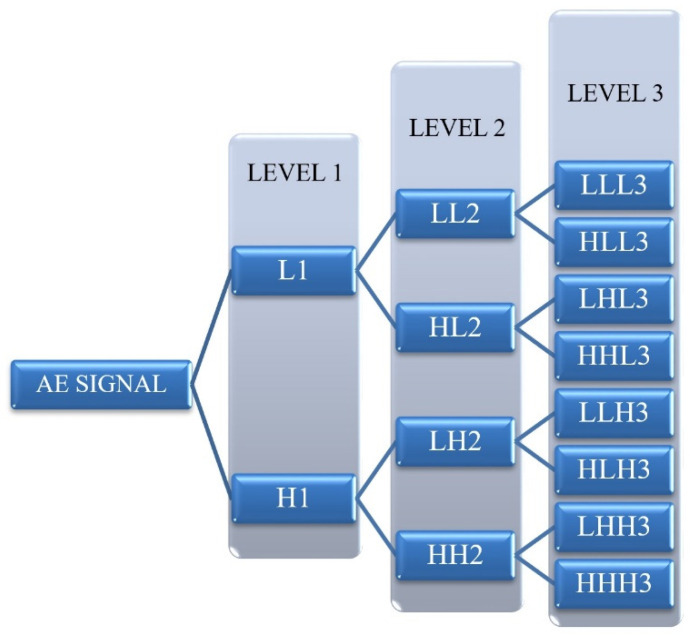
Tree diagram of Wavelet Packet Transform.

In general, wavelet transform is one of the advanced approaches which is utilized in image and signal processing. WPT is one of the main methods of wavelet transform. In this method, the AE signal is decomposed into two categories at different levels. The first category includes an approximation that contains the low frequency components, and the second category covers the detailed parts of the signal regarding the high frequency components. At each level of the WPT method, each approximate part and each detailed part of the signal are, in turn, decomposed again onto an approximate and a detailed part in the next level [19], as is schematically demonstrated in Figure 8. In this method, the frequency bandwidth of the approximate and detailed components in each level can be calculated by [20]:

(2) $$\left[\frac{1}{2}f_{s}2^{-i},\frac{1}{2}f_{s}2^{-(i-1)}\right]$$

(3) $$\left[0,\frac{1}{2}f_{s}2^{-i}\right]$$

In this case, $f_{s}$ is considered as a sample rate and the number of components at level i is $2^{i}$. The energy criterion is used to calculate the energy distribution of each component. The energy of components at each level can be calculated by:

(4) $$E_{i}^{j}(t)=\sum_{\tau =t_{0}}^{t}{\left(f_{i}^{j}(\tau )\right)}^{2}$$

where, $E_{i}^{1},\ldots ,E_{i}^{j}$ are considered as the energy of each component, and $f_{i}^{1}\;\ldots \;f_{i}^{j}$ are the components at the ith level of the signal, and the total energy of this signal can be calculated as:

(5) $$E_{Total}(t)=\sum_{i}^{}E_{i}^{j}(t)$$

Eventually, $P_{i}^{j}$ which is the relative energy distribution at each level can be assumed by:

(6) $$P_{i}^{j}(t)=\frac{E_{i}^{j}(t)}{E_{Total}(t)}\;j={1\ldots 2}^{i}$$

In this method, in order to calculate the sufficient levels of decomposition, a mathematical criterion called entropy criterion is used [21]. The third level is calculated as the suitable and adequate level in this research. 

As mentioned, sentry function is another method for analyzing the AE signals that is utilized. One of the advantages of this method is to simultaneously investigate the qualitative aspects and the mechanical information of the AE signals. Sentry function is defined as the logarithm of ratio of the strain energy and the AE energy due to damage, as shown below:

(7) $$f(x)=\ln\left[\frac{E_{s}(x)}{E_{a}(x)}\right]$$

**Figure 9:**
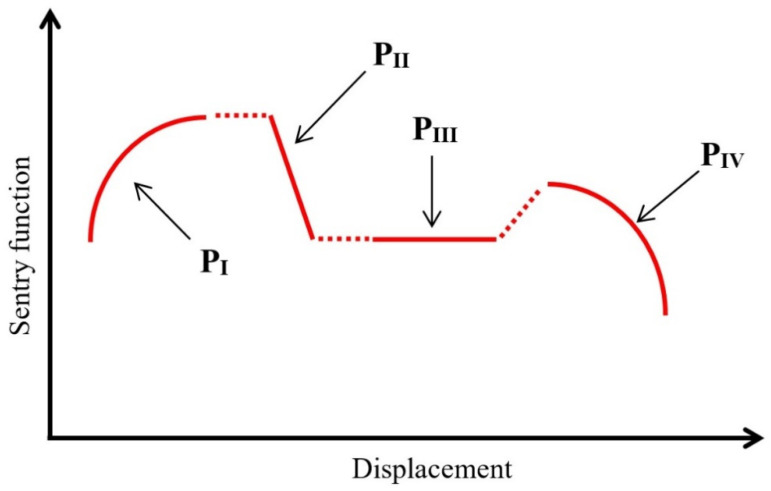
The basic functions which are utilized to describe sentry function.

In this equation, *E_s_*(*x*) is the strain energy that is considered as the area under the load–displacement curve and *E_a_*(*x*) is the AE energy released by the damage events, and *x* is the driving variable of a specific event which, in this study, is equal to the displacement of the applied load. As shown in Figure 9, the behavior of the sentry function–displacement diagram can be classified in four different categories: (1) Increasing trend, which is specified by P_I_ (*x*) and indicates during this stage no phenomenal damage occurring in the specimen. This stage is proceeded by a drop in the applied load; (2) Sudden drop, specified by P_II_ (*x*), which shows that notable damage has occurred during the test; (3) Constant behavior, specified by P_III_ (*x*), corresponded to an equivalency between the damages and some stiffening failures such as fiber bridging; (4) Gradual decrease, specified by P_IV_ (*x*), which indicates that the structure is losing its load carrying capability. The area beneath the sentry function–displacement curve can be considered as a parameter for showing the resistance of a structure against various modes of failures. A higher amount of this area shows a higher susceptibility of strain energy storing, which in turn states that this structure has less sensitive to the propagation of the damage [20,22].

## 3. Results and Discussion

**Figure 10:**
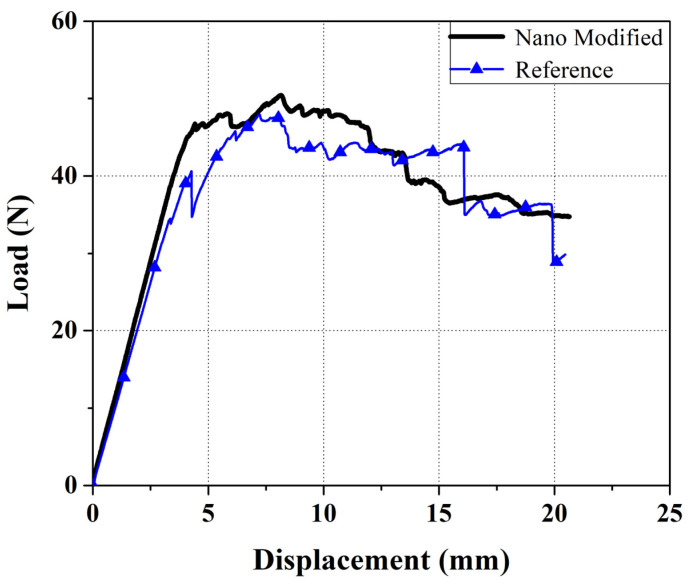
Load–displacement diagram of Mode I tests.

The load–displacement diagram of mode I that tests for both nano modified and reference samples is shown in Figure 10. It can be concluded from this diagram that taking advantage of nanofibers increases the required load for crack growth in mode I test. According to these results, the amount of critical strain energy release rates (*G_IC_*) for nanomodified and reference are 0.58 and 0.42 $MPa\sqrt{mm}$, respectively. Thus, the *G_IC_* amount of the specimen with nanofibers has increased by about 38% compared to specimen without nanofibers.

**Figure 11:**
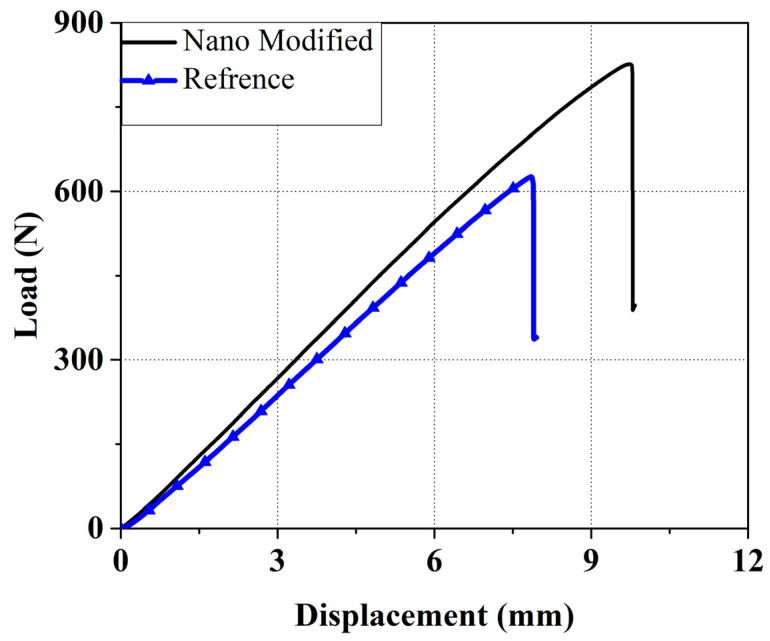
Load–displacement diagram of the Mode II tests.

The load–displacement diagram of the mode II tests is shown in Figure 11. According to this diagram, the maximum applied load for nanomodified and reference sample is about 827 and 625, respectively, and the critical strain energy release rates of the mode II test (*G_IIC_*) for the nanomodified and reference samples are 3.96 and 2.6, respectively. Thus, using nanofibers caused a 52% and 32% increase in critical strain energy release rates and maximum applied load, respectively.

**Figure 12:**
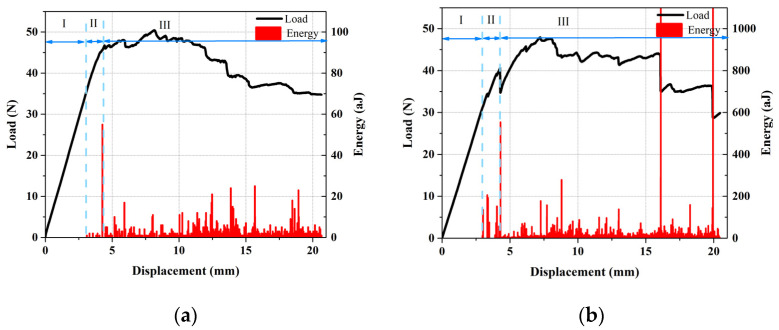
Load–displacement and AE energy–displacement diagrams of: (**a**) nanomodified and (**b**) reference specimens.

As shown in Figure 12, due to the AE signals, each of the mode I tests can be divided in to three areas. In area (I), an elastic behavior with a linear increase in load can be observed, while no sensible signals are recorded. In zone (II), some initial damages such as matrix cracking occur. Due to these initial damages, the specimen loses some stiffness, which leads to the load diagram slope decreasing, and some AE signals with a low amount of energy are recorded. Finally, in zone (III), the crack begins to propagate and all the damaged mechanisms (including matrix cracking, matrix/fiber debonding, fiber breakage, and delamination) occur. Due to these damage mechanisms, load diagram drops, and many AE signals with different energies are produced. According to Figure 12, in all three zones, including nanofiber, cause an increase in the amount of the maximum applied load.

**Figure 13:**
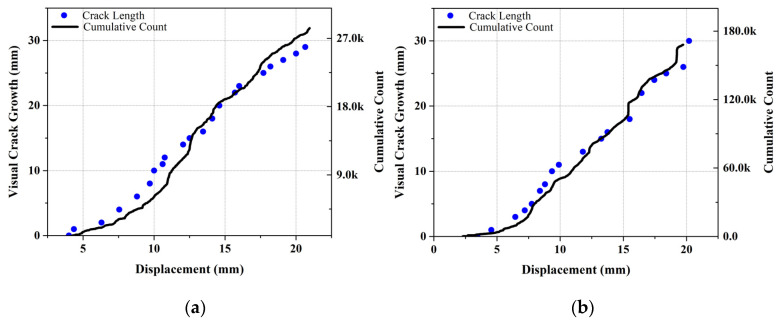
Crack length and cumulative count–displacement diagrams for: (**a**) nanomodified and (**b**) reference specimens.

In Figure 13, the crack growth and AE cumulative count–displacement diagrams for both reference and nanomodified samples can be seen. It can be concluded from these diagrams that, in both reference and nanomodified specimens, cumulative count and crack growth are related. In other words, when the crack growth rate increases, the rate of the cumulative count rises as well. Accordingly, comparing the AE cumulative counts of the two diagrams of Figure 13 reveals the fact that utilizing PCL nanofibers causes a decrease in this parameter, because nanofibers decrease the amount of damages that occur during the test.

**Figure 14:**
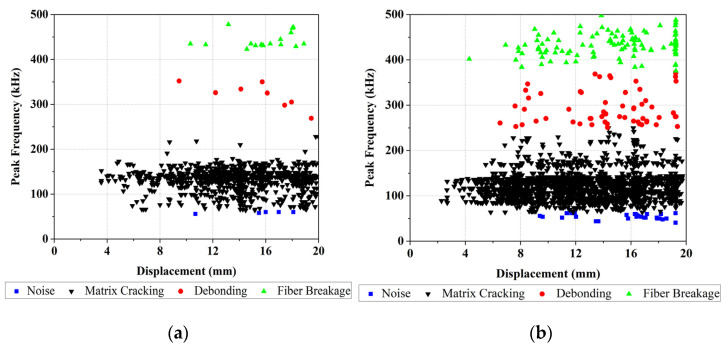
The peak frequency of acoustic emission signals for: (**a**) nanomodified, (**b**) Reference specimens.

The peak frequency of recorded AE signals (P.Freq) of both reference and nanomodified laminates tests are shown in Figure 14. In the conventional method, peak frequency is one of the AE signal parameters used for determining the type of damage. As mentioned, peak frequencies between 62.5 and 250 kHz refer to matrix cracking, between 250 and 375 kHz are related to fiber/matrix debonding, and those in the range from 375 to 500 kHz belong to fiber breakage [18]. According to this classification, for showing these damage mechanisms, each diagram in Figure 14 is classified into three zones. By considering these results, the amount of AE events of different damage mechanisms for both reference and nanomodified specimens are shown in Table 1. According to these results, in the DCB tests, applying PCL nanofibers reduces matrix cracking, matrix/fiber debonding, and fiber breakage damage mechanisms by about 75%, 94%, and 79%, respectively. As a result, PCL nanofibers causes a significant decrease in fiber/matrix debonding mechanism.

WPT is another method which is applied in this study for analyzing AE signals that are recorded during mode I tests. Here, each waveform of the AE signals is decomposed into three levels and eight wavelet components based on their frequency ranges. In order to specify the peak frequency range of each component, the Fast FFT is used. Matlab software is used in calculating and analyzing each waveform of the recorded AE signals.

**Figure 15:**
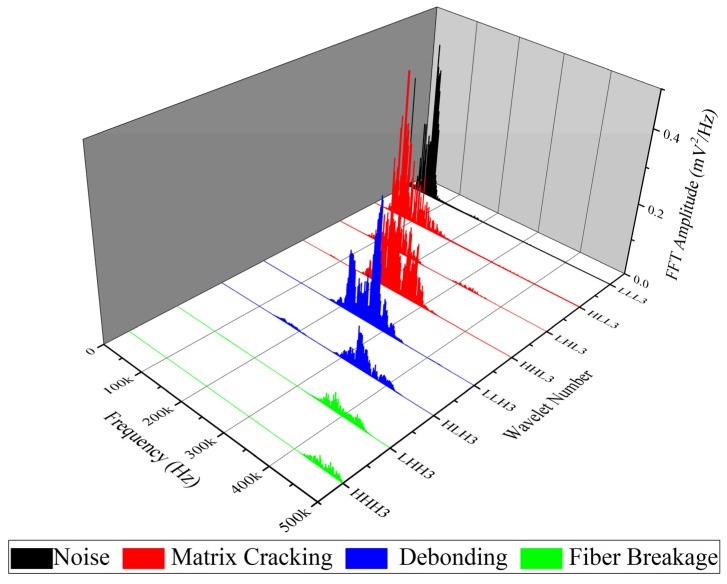
FFT of all eight components of level 3 for nanomodified specimen.

**Figure 16:**
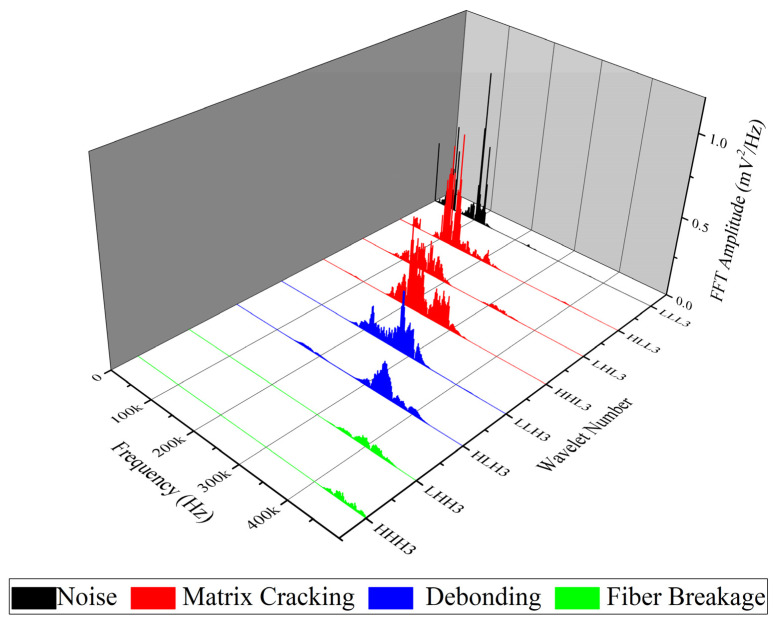
FFT of all eight components of level 3 for reference specimen.

**Figure 17:**
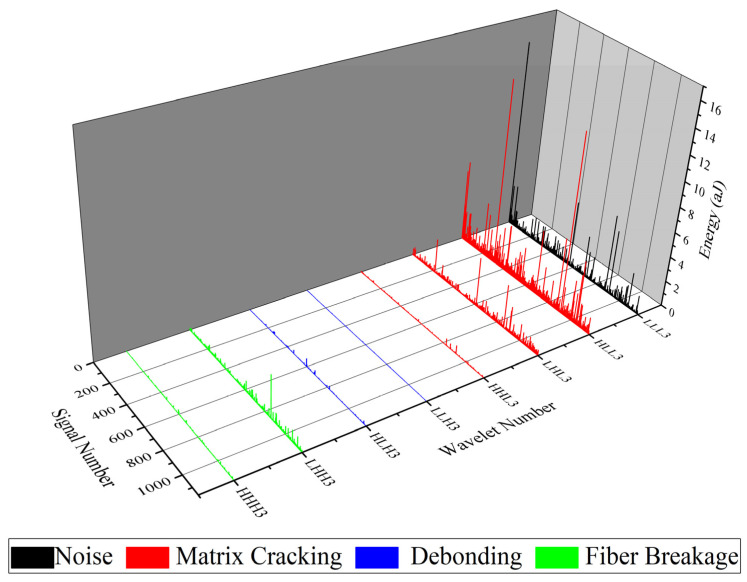
Energy distribution of all components of level 3 for nanomodified specimen.

**Figure 18:**
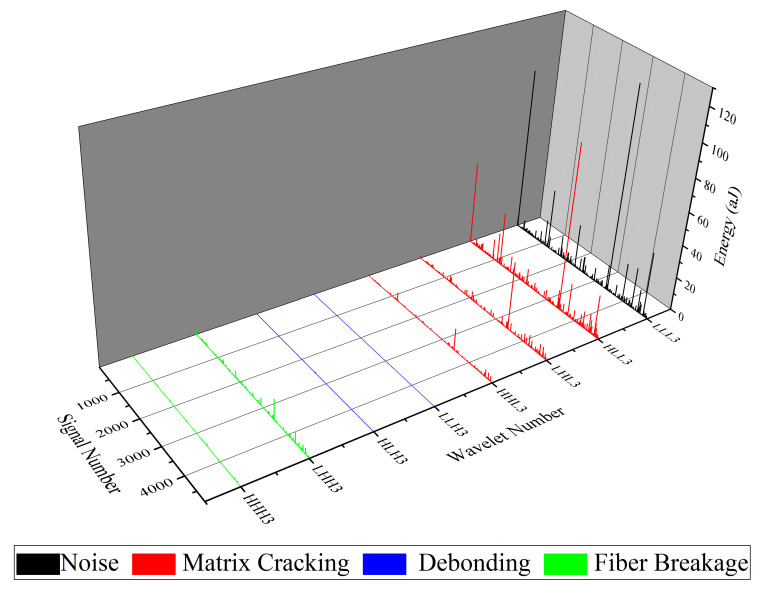
Energy distribution of all components of level 3 for reference specimen.

The FFT results for nanomodified and reference specimens are illustrated in Figure 15 and Figure 16, respectively. In both diagrams, the first component has a peak frequency between 0 and 62.5 kHz that is related to environmental noise. The second, third, and forth components have a peak frequency between 62.5 and 250 kHz, therefore these components can be interpreted as indicative of a matrix cracking failure mechanism. The fifth and sixth components are considered to be fiber/matrix debonding, since the peak frequency of these components has been located in the range of 250 to 375 kHz. The last two components have a peak frequency between 375 and 500 kHz, which are related to the fiber breakage failure mechanism. The energy of each component is then determined by means of energy criteria. The amount of energy for each component for both nanomodified and reference laminates are shown in Figure 17 and Figure 18, respectively. The quantity of each failure mechanism can be specified by considering the AE energy distribution in each damage frequency range for both specimens. The quantity of energy for each failure mechanism for both specimens, nanomodified and reference, are compared in Table 2. According to the results obtained by the WPT method, applying PCL nanofibers in mode I test causes a reduction in matrix cracking, fiber/matrix debonding, and fiber breakage failure mechanisms by 91%, 67%, and 93%, respectively.

In Table 3, the results of the PCL nanofiber’s effect on the damage mechanisms’ reduction, based on conventional (peak frequency) and WPT analyzing methods, are compared. According to these results, both methods of analysis indicate that PCL nanofibers cause a significant reduction in all three dominant failure mechanisms. However, the predicted amount of this reduction in each failure mechanism is different, based on the analyzing method.

**Figure 19:**
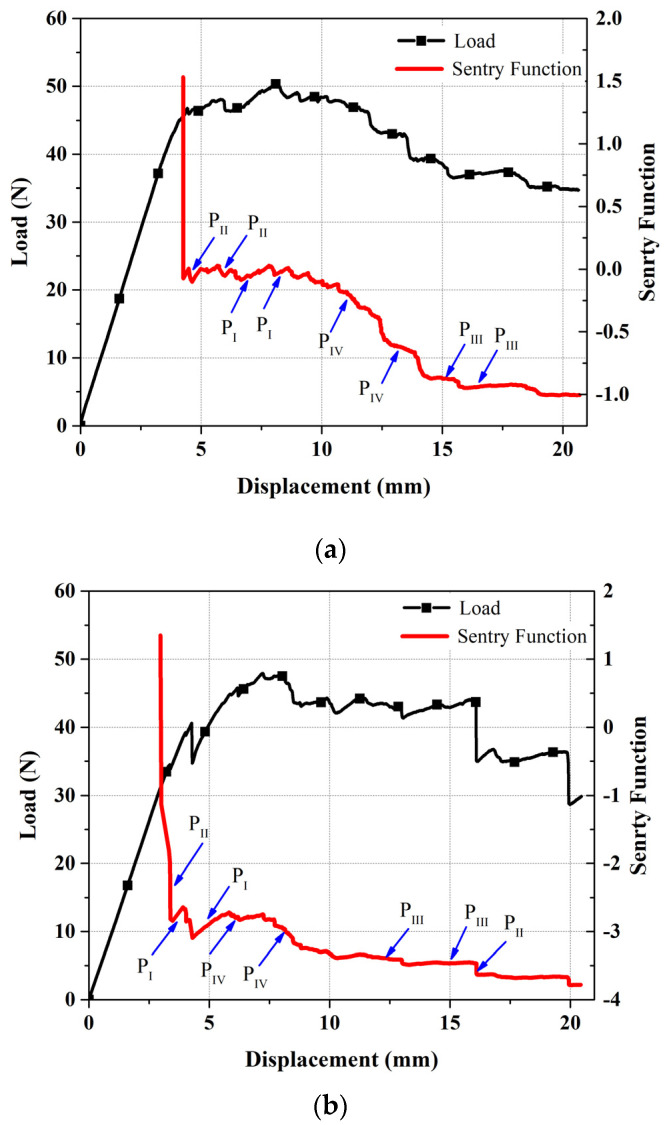
Sentry function and load diagrams for (**a**) nano modified (**b**) reference specimens.

Sentry function and load displacement diagrams for both reference and nanomodified samples are shown in Figure 19. As can be seen in these diagrams, the amount of the first sudden drop of the sentry function in the reference specimen is larger than in the nanomodified samples, which indicates that, in the reference specimen, the amount of energy due to failure mechanisms that cause primary crack propagation is more than that found in the nanomodified specimens. After the first drop in the sentry function curve, in the nanomodified sample, the range of the increasing trend (P_I_) and the constant behavior (P_III_) are larger than the reference sample, whereas the range of the sudden drop (P_II_) in reference sample is wider than in the nanomodified sample. Thus, it can be concluded that, through this zone, the nanomodified sample is more resistant to crack propagation.

**Figure 20:**
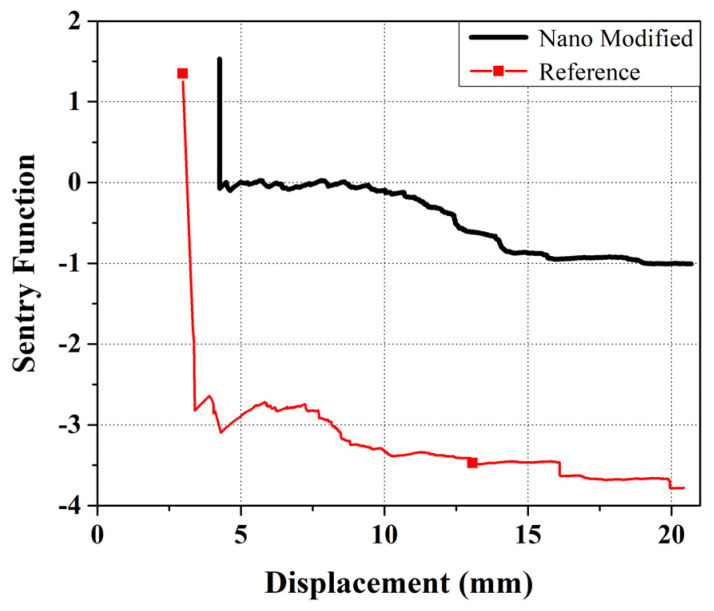
Comparison between the sentry function for the reference and nanomodified laminates.

In Figure 20, the sentry function of both nanomodified and reference samples are compared. As mentioned above, the integral of the sentry function, or, in the other words, the area under the sentry function–displacement curve, can be considered as a parameter of damage resistance capability. According to the results of Figure 20, throughout the displacement, the sentry function for the reference sample passes below the nanomodified specimen. Thus, it can be concluded that the value of the integral of the sentry function related to the nanomodified specimen is higher than the reference sample. As a result, the nanomodified sample has a higher damage resistance capability.

## 4. Conclusions

In this study, the effect of using polycaprolatone nanofibers (PCL) in mode I and mode II fracture tests is investigated. Electrospinning method is used in order to produce PCL nanofibers. Mode I and mode II tests are conducted according to the relevant standards. In the nanomodified samples, a layer of PCL nanofibers was placed in front of the primary crack. Acoustic emission (AE) analysis is utilized with the aim of investigating the effect of including nanofibers on the failure mechanisms that occur during mode I tests. In order to analyze AE signals, three methods, including the conventional method, wavelet packet transform (WPT), and sentry function, are used. The following results are concluded from this study:

According to mechanical results, using PCL nanofibers causes the critical strain energy release rate of mode I and mode II to increase by about 38% and 52%, respectively. Moreover, the maximum applied load in mode II increases by about 32% in nanomodified laminates.

According to the conventional AE analyzing method, using PCL nanofibers results in a reduction in matrix cracking, fiber/matrix debonding, and fiber breakage failure mechanisms by 75%, 94%, and 79%, respectively, in mode I test.

The WPT method shows that, in mode I tests, the specimens which are modified by PCL nanofibers are involved, with a reduction of 91%, 67%, and 93% in matrix cracking, fiber/matrix debonding, and fiber breakage, respectively.

The inclusion of PCL nanofibers causes a decrease in both the amount and number of sudden drops in the sentry function results of mode I tests. On the other hand, nanomodified specimens experience higher amounts and numbers of increasing trends and constant behaviors of the sentry function. This proves that the PCL nanomodified laminates are significantly resistant to damage and, as a result, show a higher damage resistance capability.

## Figures and Tables

**Table 1:** The amount of AE events of different damage mechanisms.

	Matrix Crecking	Debonding	Fiber Breakage
Nanomodified	1128	8	22
Reference	4586	142	106

**Table 2:** The amount of energy for each failure mechanism according to WPT method.

	Matrix Crecking	Debonding	Fiber Breakage
Nanomodified	1522.8	27.2	211.4
Reference	16,977.4	83.2	3028.1

**Table 3:** The percentage of the damage mechanisms’ reduction based on conventional and WPT methods.

	Matrix Crecking	Debonding	Fiber Breakage
Conventional	75%	94%	79%
WPT	91%	67%	93%

## Data Availability

Not applicable.
